# Silica gel with covalently attached bambusuril macrocycle for dicyanoaurate sorption from water

**DOI:** 10.3762/bjoc.21.201

**Published:** 2025-11-24

**Authors:** Michaela Šusterová, Vladimír Šindelář

**Affiliations:** 1 Department of Chemistry, Faculty of Science, Masaryk University, Kamenice 5, 625 00 Brno, Czech Republic; 2 RECETOX, Faculty of Science, Masaryk University, Kamenice 5, 625 00 Brno, Czech Republic

**Keywords:** anion binding, anion extraction, anion receptor, gold mining, macrocycles

## Abstract

Anion removal from aqueous solutions remains a major challenge due to the strong hydration of anions. Here, we report the preparation of silica gel functionalized with covalently anchored bambusuril macrocycles. In aqueous solution, this material efficiently sorbs dicyanoaurate(I), the key anion in gold mining, even in the presence of competing dicyanoargentate(I) anions. We also examine the recyclability of the material and assess its stability in organic solvents, comparing its performance with that of a previously developed material containing noncovalently bound bambusuril.

## Introduction

Inorganic anions play essential roles in a variety of biological and biochemical mechanisms and are also involved in many industrial and manufacturing processes. Due to their significant environmental impact, it is important to monitor their presence and control their concentrations, particularly in water. To maintain anion levels within acceptable ranges, their removal from the environment is often necessary [1–2].

Current technologies for removing anions from aqueous solutions include chemical coprecipitation [3], ion-exchange [4], or membrane filtration [5]. In the field of anion extraction, there is growing interest in solid-phase substrates functionalized with synthetic macrocyclic receptors. These materials employ a supramolecular approach based on specific host–guest interactions between the immobilized macrocycle and the guest in solution, often leading not only to improved extraction effectivity but also to enhanced selectivity [2]. Anion extractants based on functionalized polystyrene or silica gel typically incorporate calixarenes [6] and their analogues, calixpyrroles [7–8], azacalixarene derivatives [9], or expanded porphyrin-like sapphyrin [10].

Although synthetic anion receptors have attracted increasing attention in the field of supramolecular chemistry, there are still relatively few reports of materials that employ macrocyclic receptors to recognize and selectively bind anionic species specifically in aqueous environments. For example, an amphiphilic poly(methyl methacrylate)-based polymer modified with calix[4]pyrrole was found to form micelles in water that capture anions from caesium salts and can be precipitated from solution upon heating [11]. Similarly, a hydrogel composed of poly(vinyl alcohol) cross-linked by a “Texas-sized” molecular box has been used to extract various anions from water [12].

Another family of macrocyclic compounds suitable for anion recognition and binding are bambusurils (BUs). BUs act as very potent anion receptors in both organic solvents and water. Furthermore, their portals can be variously functionalized, for instance by carboxylic moieties [13], making them available for further reactions with different substrates. We have recently presented a material (SG-BnBU) based on silica gel containing dodecabenzylbambus[6]uril (BnBU) non-covalently bound on its surface [14]. We showed that this material is able to remove dicyanoaurate(I) anion from water, the principal cyanide-based species generated during gold mining processes. However, the use of the SG-BnBU material is problematic as BnBU can leach from the material into the solution. To address this limitation, we have developed and herein report a new material, SG-NHCO-BU1, in which the BU1 macrocycle is covalently grafted onto the surface of silica gel. We demonstrate that this hybrid material can be used for the selective dicyanoaurate(I) sorption from water. Moreover, it is shown that unlike SG-BnBU, SG-NHCO-BU1 exhibits good stability in organic solvents.

## Results and Discussion

### Decoration of silica gel with bambusuril

**Scheme 1:**
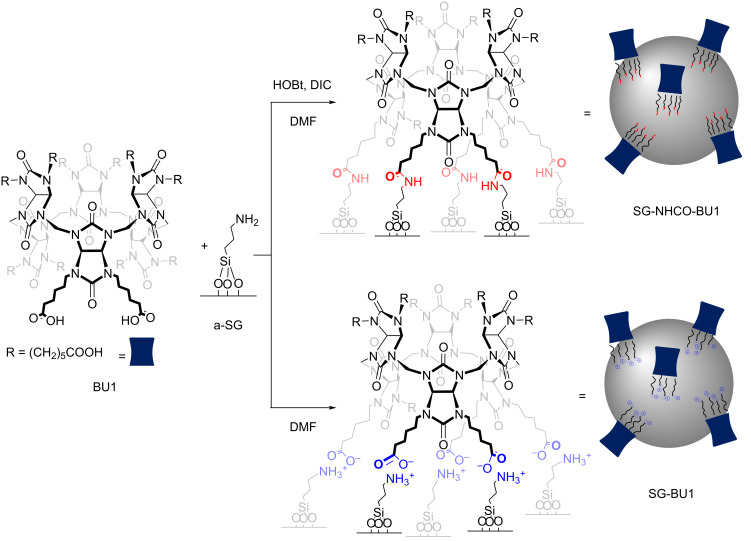
Synthesis of SG-NHCO-BU1 and SG-BU1 materials based on covalently and non-covalently attached BU1 on the SG surface.

For the modification of silica gel (SG), we selected previously reported bambus[6]uril BU1 (Scheme 1) containing 12 carboxyalkyl substituents [13]. This macrocycle was chosen for several reasons: BU1 can be attached to SG not only by electrostatic interactions but also covalently, the BU1 synthesis is straightforward lacking difficult and time-consuming purification, and it is obtained in relatively high yield over 60%. Once BU1 was prepared, its covalent attachment on the surface of SG was achieved in two steps. First, SG, chosen as the solid substrate, was reacted with an excess of 3-aminopropyltriethoxysilane (APTES) in toluene under reflux to obtain a-SG material with amino groups introduced on the SG surface [15–16]. In the second step, a-SG was treated with BU1, in the presence of 1-hydroxybenzotriazole (HOBt) and 1,3-diisopropylcarbodiimide (DIC) in DMF, resulting in SG-NHCO-BU1 with BU1 covalently attached through amide bonds (Scheme 1). The material was thoroughly washed with DMF, water, and methanol to remove byproducts and unreacted BU1. The grafting reaction was conducted using a 9:1 (w/w) ratio of a-SG to BU1. This ratio was selected based on our previously published results for the noncovalent SG-BnBU system [14]. Besides the covalent SG-NHCO-BU1 material, we also prepared the non-covalent SG-BU1 material, in which BU1 is attached on the SG surface through electrostatic interactions (Scheme 1). The latter reaction was also done using a 9:1 (w/w) ratio of a-SG to BU1, by treatment of a-SG with the macrocycle in DMF at ambient temperature (Scheme 1). The material was then isolated by filtration and washed with DMF and methanol.

The prepared a-SG, SG-NHCO-BU1, and SG-BU1 materials were characterized by FTIR spectroscopy (Supporting Information File 1, Figure S1). In comparison to crude silica gel, the diminished band corresponding to the –OH group (around 980 cm^−1^) and the appearance of new bands characteristic of amino groups (around 3000 cm^−1^) indicate the successful introduction of amino groups onto the silica gel surface, confirming the formation of a-SG. Upon modification of a-SG with BU1, additional bands characteristic of BU1 appeared. When comparing BU1 with the SG-NHCO-BU1 material, the C=O vibration band shifted from 1703 cm^−1^ to 1696 cm^−1^ indicating the formation of an amide bond. Additionally, a new absorption band at 1558 cm^−1^ was observed in the spectra of SG-NHCO-BU1 further confirming covalent attachment of BU1 through an amide bond. In the case of SG-BU1, a C=O vibrational band is observed at 1705 cm^−1^, which corresponds to the vibration of the C=O of BU1 carboxylic acid groups. Other bands corresponding to BU1 were also visible in the spectrum, confirming the presence of the macrocycle in both materials.

**Figure 1:**
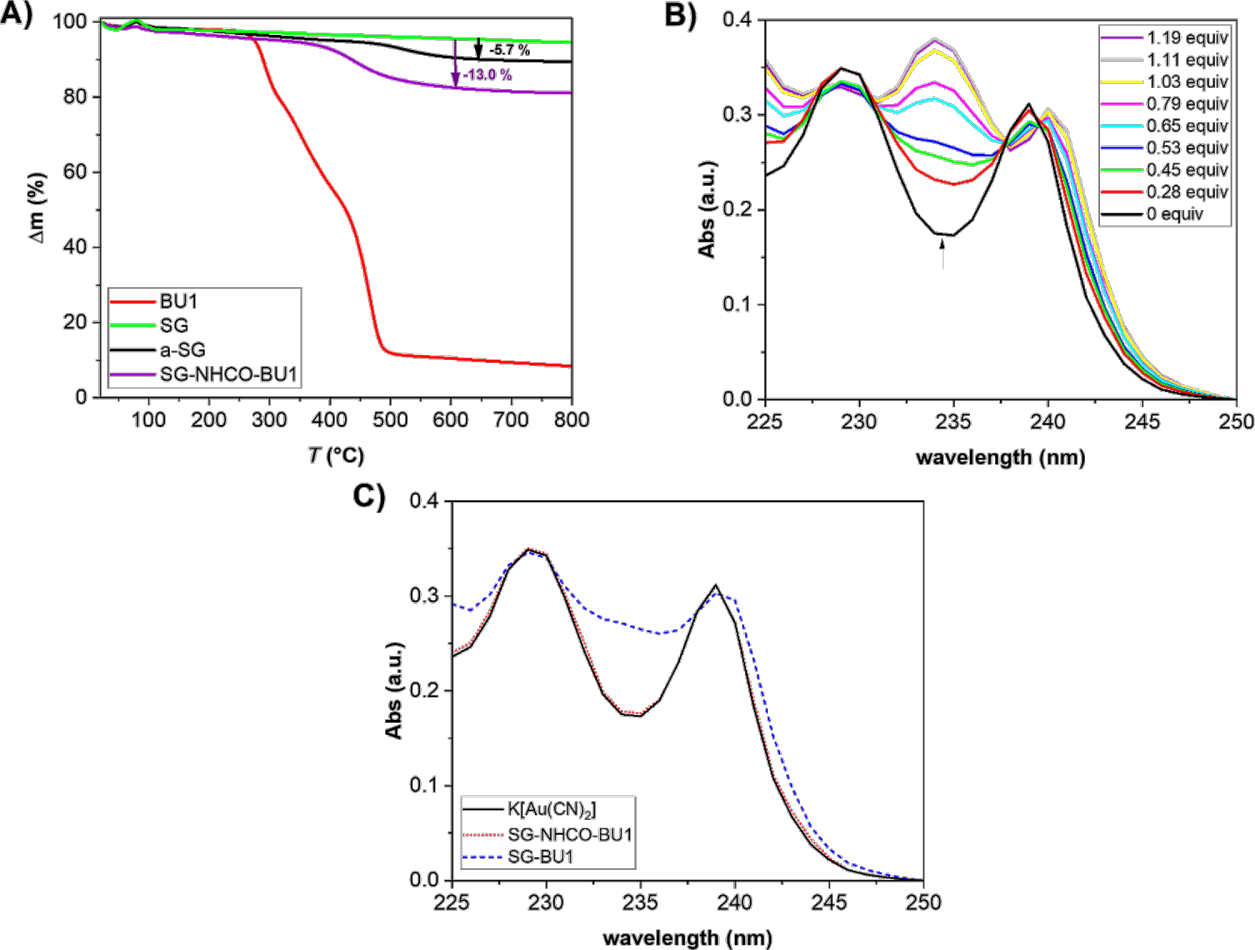
A) Thermogravimetric analyses of BU1, SG, a-SG, and SG-NHCO-BU1. B) UV–vis titration of K[Au(CN)_2_] (0.5 mM) with increasing amount of BU1. C) UV–vis spectra of K[Au(CN)_2_] (0.5 mM) before and after additions of supernatants obtained by treatment of SG-NHCO-BU1 and SG-BU1 with aqueous 1 M KOH.

The attachment of APTES and subsequently BU1 on SG was further confirmed by thermogravimetric analysis (TGA) (Figure 1A). Pure SG is highly thermally stable, undergoing only minimal decomposition at temperatures below 800 °C as confirmed by the analysis. In contrast, a-SG, SG-NHCO-BU1, and SG-BU1 exhibited reduced thermal stability due to the presence of organic substituents (Figure 1A and Supporting Information File 1, Figure S1C). A weight loss of 10% for BU1, SG-NHCO-BU1, and SG-BU1 was observed at 289 °C, 427 °C, and 438 °C.

Additionally, TGA allowed determination of the content of BU1 grafted on the surface in SG-NHCO-BU1 and SG-BU1. As stated above, the material was prepared by mixing of a-SG to BU1 in a 9:1 (w/w) ratio. The analysis revealed that there is approximately 7.3 wt % of BU1 in SG-NHCO-BU1, which corresponds to 74% of the used BU1 attached during its reaction with a-SG (Figure 1A). SG-BU1 contained 7.7 wt % of BU1 which is comparable with the material of covalently attached BU1 (Supporting Information File 1, Figure S1). Thus, according to the TGA, SG-NHCO-BU1 contains 73 mg of BU1 per 1 g of material, which corresponds to 0.032 mmol of BU1 per gram and SG-BU1 comprises 0.034 mol of BU1 per gram.

In order to prove that BU1 is bound through amide bonds on the substrate and it does not leach into solution, we have treated the SG-NHCO-BU1 material with 1 M KOH, followed by addition of dicyanoaurate ([Au(CN)_2_]^−^) to the supernatant. It was shown previously that the absorption spectrum of dicyanoaurate in aqueous solution changes upon formation of an inclusion complex between the anion and a bambusuril macrocycle [17]. A solution of dicyanoaurate in 1 M KOH was titrated by BU1 and the changes in the absorption spectra of the anion were in agreement with those previously reported in pure water (Figure 1B). Dicyanoaurate absorption bands with maxima at 239 and 229 nm decreased in their intensity in the presence of BU1, while a new band appeared at 234 nm. As the complex formation is almost quantitative at millimolar concentrations (association constant is 1.3 × 10^6^ M^−1^ in water) [17], dicyanoaurate can be utilized for the determination of the BU1 content in solution in case BU1 leaches from the substrate. For comparison, SG-BU1 was treated in the same way. After the treatment of the SG-NHCO-BU1 material with 1 M KOH, the supernatant was analyzed in the presence of 1 mM dicyanoaurate by UV–vis spectroscopy (Figure 1C). The measured spectra were identical with the spectrum of pure dicyanoaurate solution revealing that SG-NHCO-BU1 did not release any BU1 and therefore, the macrocycle is indeed covalently bound in the SG-NHCO-BU1 material. This is in contrast with the SG-BU1 material, which released BU1 to the KOH solution as evidenced by marked changes of dicyanoaurate in its absorption spectra (Figure 1C).

A potential application of these materials depends on their stability in different solvents. In terms of practical implementation, BU must stay attached to the solid substrate and should not leach into solution upon usage; for example, as shown above, SG-BU1 has limitations to be used in basic environments. To investigate further the stability of the prepared materials, we compared the behavior of SG-NHCO-BU1, SG-BU1, and SG-BnBU in different solvent systems (Supporting Information File 1, Figure S3). Chloroform, dichloromethane, and methanol were investigated due to their frequent use as mobile phases in liquid chromatography; aqueous phosphate buffer (pH 7.1) was chosen to test the materials’ stability in an aqueous environment. ^1^H NMR measurements showed that neither material released BU into organic solvents in the absence of anions. This was unexpected for SG-BnBU, since BnBU is readily soluble in chloroform and dichloromethane and is only physically adsorbed on SG. However, upon addition of chloride anions, either as tetrabutylammonium (TBA) or sodium salt, BnBU formed a complex with the anion and dissolved in chloroform and dichloromethane and even in methanol. Thus, SG-BnBU remained stable only in water in the absence and presence of salt showing it is suitable for applications in an aqueous environment. SG-BU1 proved to be stable in chloroform, dichloromethane, and methanol even after addition of TBACl or NaCl, respectively. We did not detect any release of BU1 from SG-BU1 in aqueous phosphate buffer by NMR, but we observed leaching of BU1 into solution upon addition of NaCl (Supporting Information File 1, Figure S4) to this solvent system. As expected, SG-NHCO-BU1 with covalently bound BU1 remained stable in all tested solvents in the absence as well as in the presence of salts.

### Anion sorption by SG-NHCO-BU1

**Figure 2:**
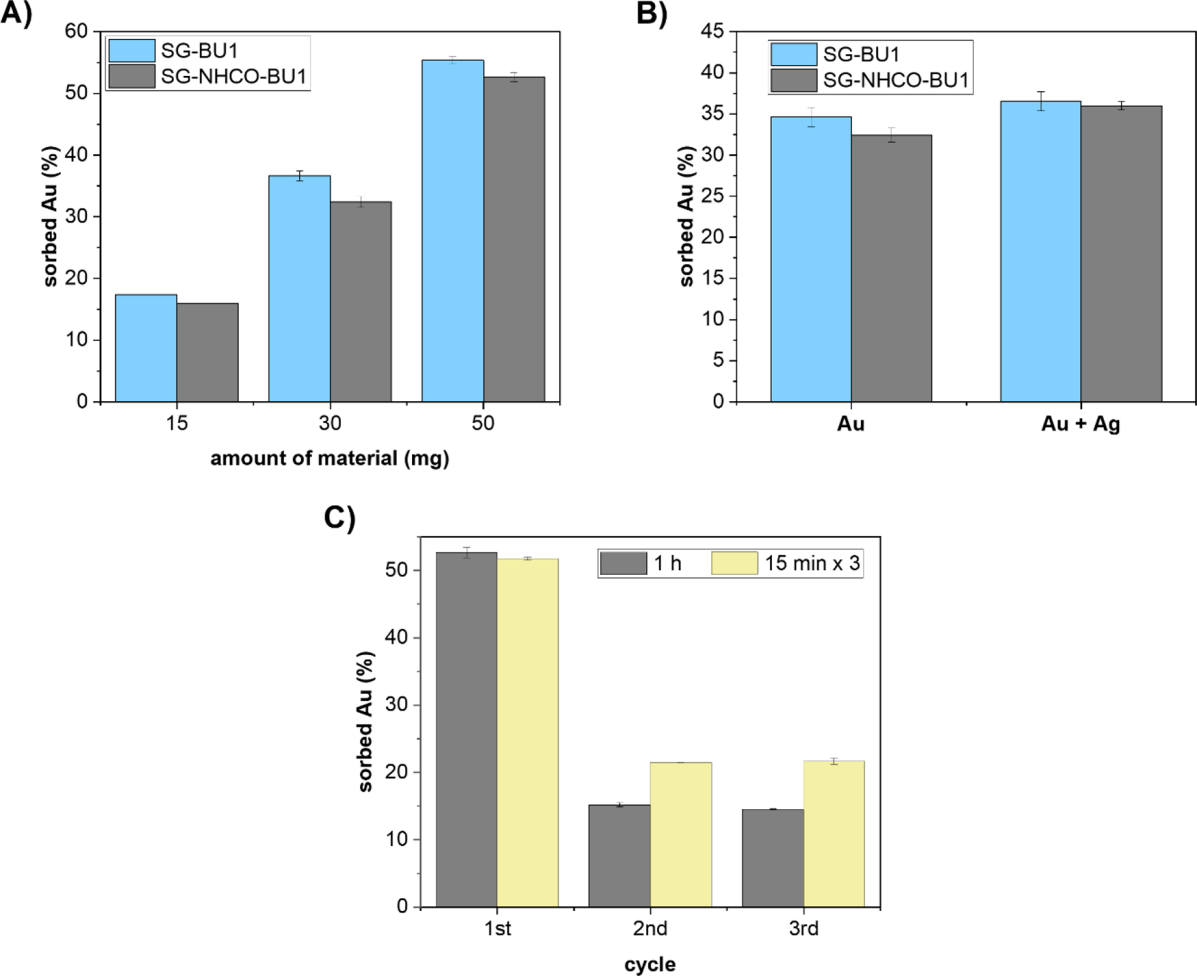
The efficiency of materials (blue SG-BU1, grey SG-NHCO-BU1) in sorbing dicyanoaurate from its water solution (1 mM, 3 mL). A) Depending on the amount of the used material. B) Measured in the absence (Au) and in the presence of K[Ag(CN)_2_] (Au + Ag); experiments were done using 30 mg of the materials. C) Dicyanoaurate sorption efficiency before (1st cycle) and after (following cycles) washing with NaBr solution (10 mM) for 1 h or 3 × 15 min; experiments were done using 50 mg of SG-NHCO-BU1.

Silica gel modified either with covalently or noncovalently immobilized BU1 was further investigated for its ability to sorb anions from water and in terms of recyclability. For this purpose, dicyanoaurate ([Au(CN)_2_]^−^) was selected as this anion can be monitored by UV–vis spectroscopy (Figure 2) and plays a central role in gold mining industry [18]. In addition, it allows comparison with the previously prepared SG-BnBU material with noncovalently bound BnBU, which was also tested using the same anion. When either SG-NHCO-BU1 or SG-BU1 was treated with a 1 mM solution of K[Au(CN)_2_] in water, the anion concentration in solution reached equilibrium after 15 minutes, consistent with the equilibration time previously established for the non-covalent system SG-BnBU (Supporting Information File 1, Figure S5). Therefore, all subsequent sorption experiments were performed with an equilibration time of 15 minutes.

Next, we evaluated the efficiency of the SG-NHCO-BU1 material in sorbing dicyanoaurate. First, we showed that SG-BU1 is stable under the experimental conditions (Supporting Information File 1, Figure S5C) and next, we investigated both materials for dicyanoaurate sorption. Thus, we gradually increased the amount of SG-NHCO-BU1/SG-BU1 in the 1 mM water solution of K[Au(CN)_2_] and observed an uptake of dicyanoaurate based on the changes in the absorption spectra of K[Au(CN)_2_]. Assuming the molar content of BU1 in the SG-NHCO-BU1/SG-BU1 materials is 0.032/0.034 mmol g^−1^ (based on TGA), we calculated that 1.3/1.4 equivalents of BU1 on the material are required to bind a single dicyanoaurate anion. A similar macrocycle-to-anion ratio was previously determined for the SG-BnBU material. In contrast, water-soluble BU and dicyanoaurate have been reported to form complexes of 1:1 stoichiometry, exhibiting near-quantitative binding at millimolar concentrations [17]. The lower binding efficiency of BUs attached to the material compared to the free macrocycle in solution can likely be attributed to the heterogeneous nature of the solid-phase system. Moreover, the anion binding ability of BU1 may be affected by its long aliphatic arms terminated with carboxylic groups, whose flexibility can enable intramolecular complexation competing for an anion binding [13].

Bambusurils are known to bind a wide range of anions with varying selectivity. Dicyanoaurate is particularly relevant to the gold mining industry, whereas dicyanoargentate is considered a contaminant in this context. To assess selectivity, we measured the sorption of dicyanoaurate from a 1 mM aqueous solution both in the absence and in the presence of an equimolar amount of dicyanoargentate. The measurements revealed that dicyanoaurate uptake was similarly high in both experiments – in the absence and in the presence of the silver salt (Figure 2B) for both materials. The minimal effect of dicyanoargentate on dicyanoaurate sorption aligns with previous findings that BU binds the former anion over 100-fold more weakly in aqueous solution [17]. The finding is also in agreement with our reported results on the noncovalent SG-BnBU system, which showed significantly higher sorption efficiency towards dicyanoaurate over dicyanoargentate [14].

We next examined the recyclability of the materials. Only SG-NHCO-BU1 was used in these experiments as SG-BU1 is not stable under the experimental conditions as the complex of BU1@[Au(CN)_2_]^−^ was leached into the solution (Figure S6, Supporting Information File 1). After the first sorption, SG-NHCO-BU1 was washed with an excess of aqueous NaBr solution (10 mM, 3 mL, 1 h) to replace [Au(CN)_2_]^−^ in the BU1 cavity with Br^−^ (Figure 2C). This washing procedure was repeated three times, and the sorption efficiency after each cycle was determined. A decrease in the uptake efficiency was observed after the first washing, but the efficiency remained preserved in following cycles. Prior to the first sorption cycle, the macrocycle is anion-free, whereas in later cycles, it remains occupied by bromide ions due to NaBr treatment, which explains differences during the first and following cycles. We further optimized the regeneration procedure by replacing a single long wash (1 hour) with multiple shorter washes (3 × 15 minutes). The uptake efficiency of SG-NHCO-BU1 improved from 15% to 21% (Figure 2C). Furthermore, the ability of SG-NHCO-BU1 to capture anions was very well in accordance to how much of dicyanoaurate was released – the amount of captured dicyanoaurate corresponded to its released amount during previous washing step (Figure S6B, Supporting Information File 1).

## Conclusion

In summary, we have developed organic–inorganic hybrid materials for anion extraction. Through a two-step modification of silica gel, two materials were obtained: SG-NHCO-BU1, in which the BU1 macrocycle is covalently attached to the silica surface via amide bonds, and SG-BU1, where the macrocycle is bound by electrostatic interactions. Both materials proved to be an efficient extractant of dicyanoaurate anions from water, achieving maximum sorption within 15 minutes. The covalent attachment of BU1 enables the material to be recycled using NaBr solution, while the SG-BU1 material disintegrates under similar conditions. We also showed that both SG-NHCO-BU1 and SG-BU1 are stable in organic solvents including chloroform, dichloromethane, and methanol even after addition of halide salts. This stability stands in contrast to previously published SG-BnBU, which contains noncovalently immobilized BnBU and undergoes macrocycle loss in all of three tested organic solvents after addition of halide salts. These results illustrate that the choice of material for anion sorption depends on the specific experimental conditions. While SG-BnBU and SG-NHCO-BU1 are suitable for dicyanoaurate sorption from aqueous solutions, SG-NHCO-BU1 and SG-BU1 could be used for anion extraction in organic media.

## Experimental

### Materials and instruments

Silica gel (Silikagel 60 (0.015–0.040 mm) for column chromatography, product number 1.15111) was dried in vacuo at 50 °C overnight before use. 3-Aminopropyltriethoxysilane (APTES), 1-hydroxybenzotriazole (HOBt), 1,3-diisopropylcarbodiimide (DIC), potassium dicyanoaurate, potassium dicyanoargentate, sodium chloride, tetrabutylammonium chloride and sodium bromide were purchased from Merck and used without further purification. Dodecakis(5-carboxypentyl)bambus[6]uril (BU1) was synthesized according to a previously published procedure [13].

Infrared spectra were recorded with an Alpha spectrophotometer (Bruker). Thermogravimetric analyses were done with a Netzsch STA 449C Jupiter and carried out in nitrogen atmosphere. The dicyanoaurate concentration in solution was determined according to UV–vis spectra recorded with a CARY 60 spectrophotometer (Agilent Technologies). NMR spectra were recorded on a Bruker Avance III 300 spectrometer (300.15 Hz, 298.15 K).

### Preparation of SG-NHCO-BU1

Silica gel (8.01 g) was dispersed in toluene (24 mL) and stirred under argon atmosphere, while heated up to 115 °C. Then, APTES (6 mL) was added, and the dispersion was stirred under inert atmosphere at elevated temperature for 5 h. After cooling, the dispersion was filtered, washed thoroughly with toluene, ethanol and acetone to remove unreacted APTES and dried in vacuo (50 °C) overnight in order to get a-SG [15–16]. Further, to prepare covalent SG-NHCO-BU1, a-SG (0.9 g) was suspended in DMF (15 mL) and shaken at ambient temperature for 30 min. BU1 (0.1 g, 1 equiv) was dissolved in a minimum amount of DMF and a solution of HOBt (12 equiv) in DMF was added, followed by addition of DIC (12 equiv). Then, the activated BU1 was added to the suspension of a-SG. The reaction was performed under Ar atmosphere for 5.5 h at ambient temperature under constant shaking (180 rpm). The resulting material was filtered and thoroughly washed with DMF, water and methanol in order to remove byproducts and unreacted BU1. After air-drying, SG-NHCO-BU1 was obtained.

### Preparation of SG-BU1

For the preparation of SG-BU1 with electrostatically bound BU1 on the surface of silica gel, a-SG (0.9 g) was suspended in DMF (15 mL) and BU1 (0.1 g), dissolved in a minimum amount of DMF, was added. The dispersion was mechanically shaken (180 rpm) at ambient temperature for 5.5 h. Afterwards, the dispersion was filtered, washed with DMF (5×) and methanol (5×) to remove unattached BU1. After air-drying in a hood overnight at ambient temperature, SG-BU1 was obtained.

### Sorption experiments with SG-NHCO-BU1

The potential of SG-NHCO-BU1 to bind anions from solution was investigated by UV–vis spectroscopy. SG-NHCO-BU1 or SG-BU1 was placed in a vial and anion solution (1 mM, 3 mL) was added. The system was shaken for 15 min at 250 rpm. Afterwards, SG-NHCO-BU1 or SG-BU1 was left to settle and the supernatant was analyzed. The actual concentration of the anion left in the solution was calculated from a calibration curve.

## Supporting Information

File 1 Characterization of materials and additional experiments regarding anion uptake.

## Data Availability

All data that supports the findings of this study is available in the published article and/or the supporting information of this article.
