# Supplementary material for: Silica gel with covalently attached bambusuril macrocycle for dicyanoaurate sorption from water
Source: Beilstein J Org Chem. 2025 Nov 24;21:2604–11. doi: 10.3762/bjoc.21.201 (PMC12667728; doi:10.3762/bjoc.21.201)
Supplement: File 1 — Characterization of materials and additional experiments regarding anion uptake. [file Beilstein_J_Org_Chem-21-2604-s001.pdf]

BEILSTEIN JOURNAL OF ORGANIC CHEMISTRY

Supporting Information

for

Silica gel with covalently attached bambusuril macrocycle for dicyanoaurate sorption from water

Michaela Šusterová and Vladimír Šindelář

Beilstein J. Org. Chem. **2025,** *21,* 2604–2611. doi:10.3762/bjoc.21.201

Characterization of materials and additional experiments regarding anion uptake

License and Terms: This is a supporting information file under the terms of the Creative Commons Attribution License (https://creativecommons.org/licenses/by/4.0). Please note that the reuse, redistribution and reproduction in particular requires that the author(s) and source are credited and that individual graphics may be subject to special legal provisions.
The license is subject to the *Beilstein Journal of Organic Chemistry* terms and conditions: (https://www.beilstein-journals.org/bjoc/terms)

Table of contents

1. Characterization of the materials S2
2. Sorption experiments S6
3. References S8

1. Characterization of the materials

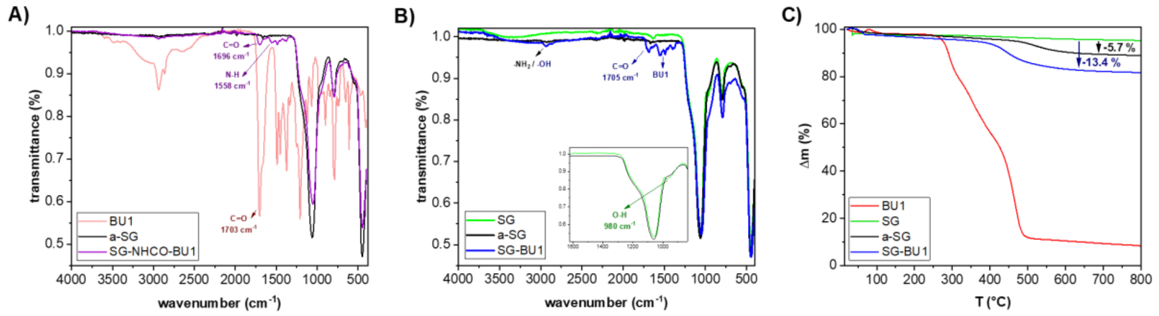

Figure S1. Comparison of ATR spectra of A) BU1, a-SG and SG-NHCO-BU1, and B) SG, a-SG and SG-BU1. C) Thermogravimetric analyses of BU1, SG, a-SG and SG-BU1.

UV–vis titration and hydrolysis

To test the stability of SG-NHCO-BU1 and SG-BU1, and eventually to quantify the amount of BU1 attached to the a-SG in SG-BU1, a treatment of the materials with KOH was conducted. SG-NHCO-BU1 or SG-BU1 was placed in a vial and treated with a solution of KOH (1 M) for 90 min under shaking. Supernatants were then separated from the solid and used for the titration with dicyanoaurate solution. Dicyanoaurate absorbs at 239 and 229 nm and our previous research showed that there is a formation of a new absorption band upon complexation of BU with dicyanoaurate, which appears at 234 nm [1]. According to a UV–vis titration of dicyanoaurate with BU1, the calibration curve may be created and used for calculation of the concentration of BU1@$[Au(CN)_2]^-$ in solution. Thus, this method allows to determine the amount of BU1 released into the solution after hydrolysis.

In the case of SG-BU1, UV–vis titration showed that ≈84% of BU1 added to the reaction with a-SG was successfully attached (i.e. released) to the material, which is in a relatively good agreement with the results obtained from TGA (77%).

The solid SG-NHCO-BU1 after treatment with KOH and washing with water was used for uptake of dicyanoaurate from its aqueous solution. The measurements showed that SG-NHCO-BU1 was able to capture anions with an efficiency of 33%, which corresponds to the amount of BU1, which is supposed to be present in the material according to the TG analysis.

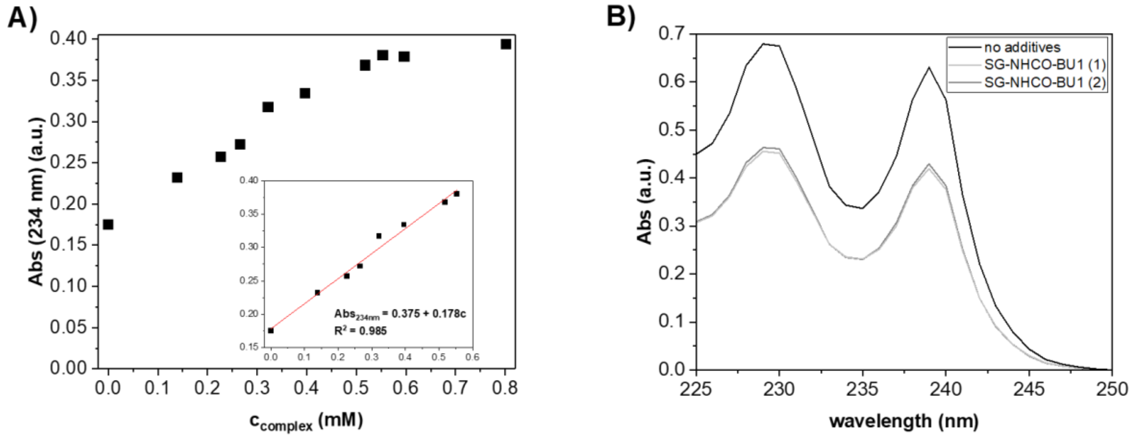

Figure S2. A) Dependency plot absorption (234 nm) vs concentration of complex (BU1⊂dicyanoaurate) with cut-off of calibration curve. B) UV–vis spectra of $K[Au(CN)_2]$ (1 mM) in the absence (no additives) and in the presence of SG-NHCO-BU1 (30 mg) after its treatment with KOH.

Stability of the material in various solvents

To monitor the stability of SG-NHCO-BU1, SG-BU1, and SG-BnBU in different solvents, ^{1}H NMR experiments were conducted. SG-NHCO-BU1, SG-BU1, or SG-BnBU was placed in an NMR tube and deuterated solvent ($CDCl_3$, CD_2Cl_2, CD_3OD, D_2O (pH 7.1)) was added. After one hour, the ^{1}H NMR spectrum was recorded to check whether any BU had been released from the material. Subsequently, a chloride salt was added to the tube, and another ^{1}H NMR spectrum was recorded. Due to the insolubility of NaCl in chloroform and dichloromethane, TBACl was chosen for these two solvents.

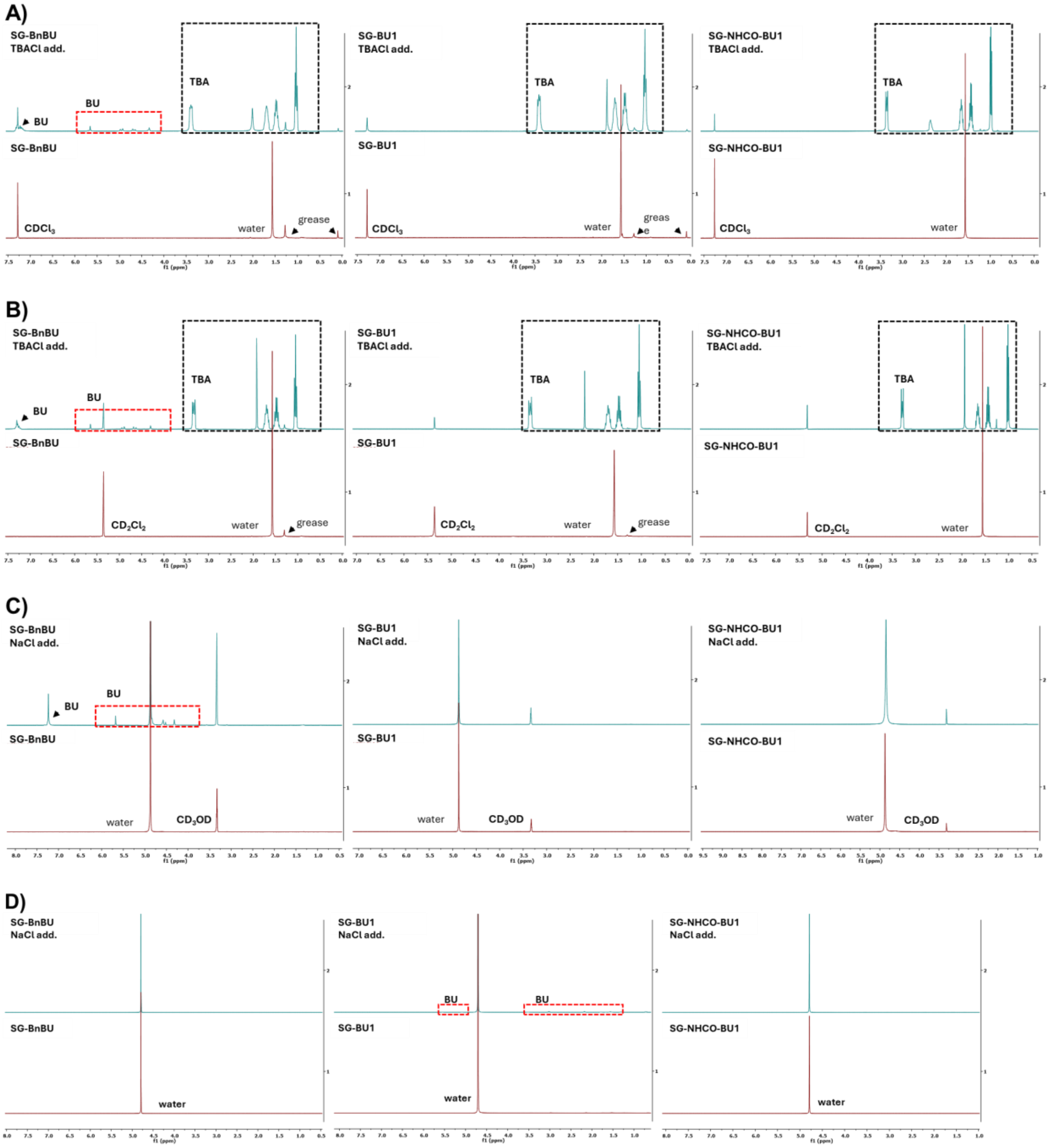

Figure S3. ^{1}H NMR spectra of SG-BnBU (left), SG-BU1 (middle), and SG-NHCO-BU1 (right) before and after anion addition in A) chloroform-*d*. B) Dichloromethane-d$_2$. C) Methanol-*d*$_4$. D) D_2O (30 mM K_2DPO_4, pH 7.1).

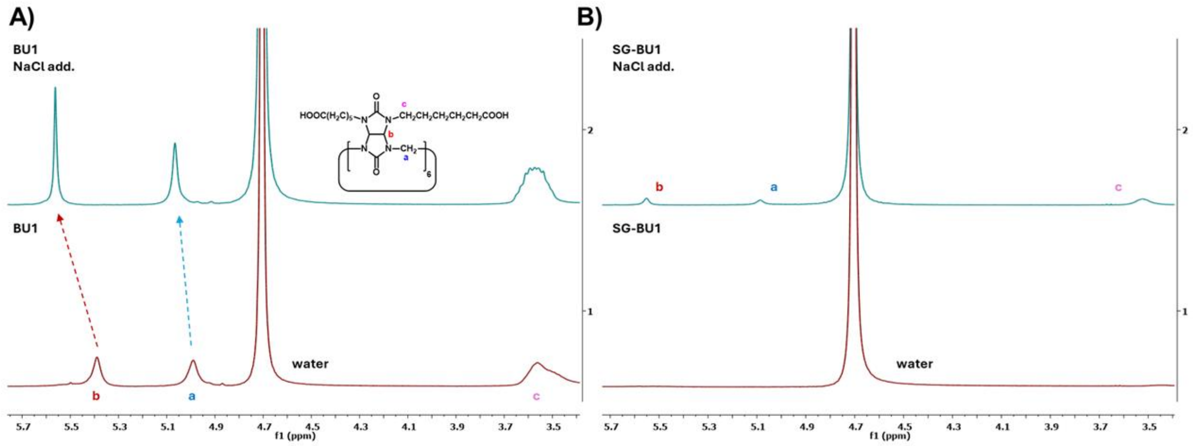

Figure S4. A) ^{1}H NMR spectra of BU1 in D_2O (30 mM K_2DPO_4, pH 7.1) before and after the addition of chloride. B) Cut-off of ^{1}H NMR spectra of SG-BU1 in D_2O (30 mM K_2DPO_4, pH 7.1) before and after chloride addition showing signals of released BU1.

2. Sorption experiments

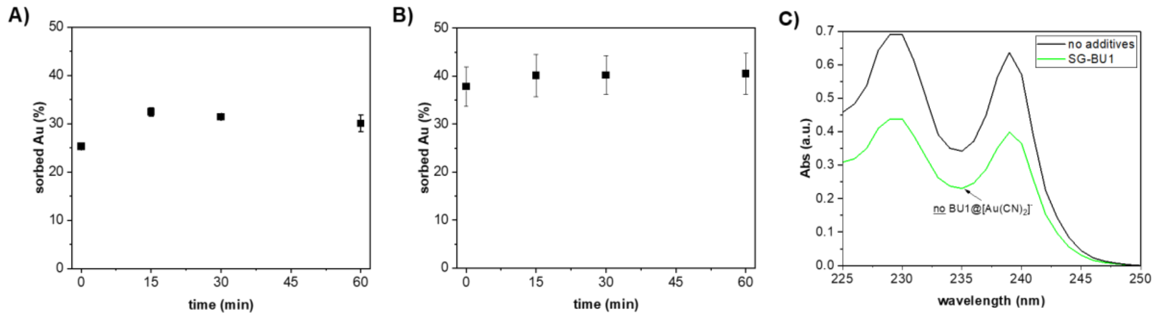

Figure S5. Time dependence of K[Au(CN)$_2$] sorption by A) SG-NHCO-BU1 and B) SG-BU1 (30 mg of material in 3 mL of 1 mM K[Au(CN)$_2$] solution). C) UV–vis spectra of K[Au(CN)$_2$] (1 mM) in the absence (no additives) and in the presence of SG-BU1 (30 mg) in milliQ water. The spectra showed the absence of absorption bands for the BU1@[Au(CN)$_2$]$^-$ complex, which indicates that BU1 does not leach from SG-BU1 to the solution.

Recycling of SG-NHCO-BU1

SG-NHCO-BU1 or SG-BU1 (30 mg), after the first dicyanoaurate uptake cycle (1 mM, 3 mL), was washed with NaBr solution (10 mM, 3 mL, 1 h). Subsequently, the materials were treated with the dicyanoaurate solution again and the uptake effectivity was monitored over several subsequent cycles. The supernatants after washing with bromide were checked by UV–vis spectroscopy in order to monitor the release of dicyanoaurate from the material (Figure S6A). The supernatant obtained after washing SG-BU1 showed absorption bands characteristic for the BU1@$[Au(CN)_2]^-$ complex, revealing that the macrocycle leaches from the SG-BU1 into the solution.

Additionally, the regeneration procedure has been modified – 50 mg of SG-NHCO-BU1 were used for dicyanoaurate uptake from its aqueous solution (1 mM, 3 mL) and then treated with NaBr (10 mM, 3 mL) for one hour or three times for 15 minutes. Afterwards, the material was treated with the dicyanoaurate solution again. The uptake efficiency as well as the amount of released dicyanoaurate upon washing were monitored.

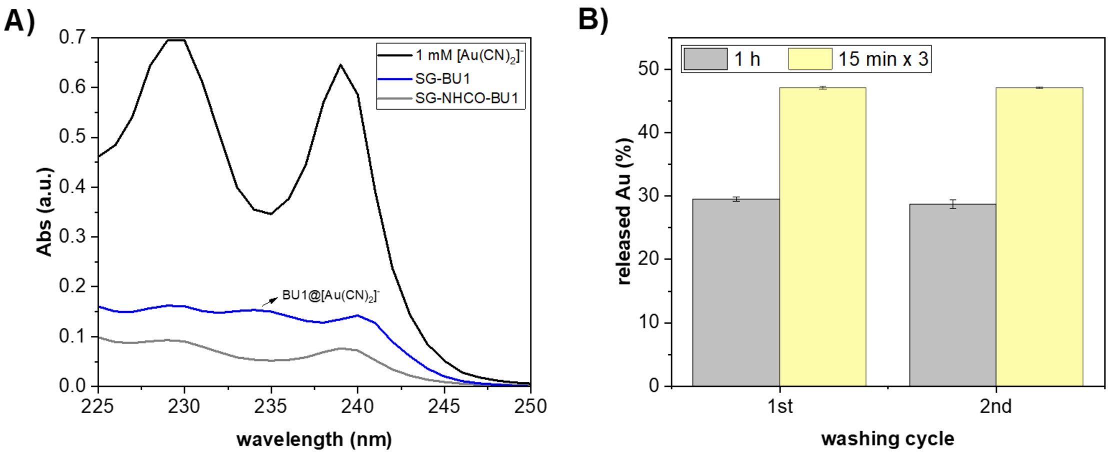

Figure S6. A) Dicyanoaurate aqueous solution (1 mM) and supernatants after washing of SG-NHCO-BU1 and SG-BU1 (30 mg) with bromide (1 h). B) Dicyanoaurate recovery efficiency from SG-NHCO-BU1 (50 mg) during the two regeneration cycles.

3. References

(1) Rando, C.; Vázquez, J.; Sokolov, J.; Kokan, Z.; Nečas, M.; Šindelář, V. *Angew. Chem. Int. Ed.* **2022**, *61*, e202210184. doi:10.1002/anie.202210184
